# Supplementary material for: Deterministic full-scenario analysis for maximum credible earthquake hazards
Source: Nat Commun. 2023 Oct 19;14:6600. doi: 10.1038/s41467-023-42410-3 (PMC10584816; doi:10.1038/s41467-023-42410-3)
Supplement: Supplementary file 1 — Supplementary Information [file 41467_2023_42410_MOESM1_ESM.pdf]

Supplementary Information for

Deterministic full-scenario analysis for maximum credible earthquake hazards

Xiang-Chao Wang[1], Jin-Ting Wang[1]*, Chu-Han Zhang[1]

[1]Department of Hydraulic Engineering, Tsinghua University, Beijing 100084, China

Corresponding author: Jin-Ting Wang (wangjt@tsinghua.edu.cn)

Model description

To simulate the seismic wave propagation of source-path-site, a three-dimensional (3D) numerical model for the vicinity of the Xiluodu dam site is constructed with the high-resolution topography and realistic velocity structures. The topography is considered using the digital elevation model data with a resolution of 30 m. Since this study only focuses on ground motions at the engineering site, accurate local velocity structures are of great importance for generating realistic ground motions there[1]. In this study, we construct a 3D numerical model (Fig. S1) by coupling the regional velocity model, East Asia EARA2014, with the geological exploration information at the dam site. Here it should be noted that the foundation conditions are very demanding for the arch dams due to their structural characteristics. Therefore, the engineering site of the Xiluodu arch dam is chosen on the hard bedrock where the minimum shear wave velocity is about 2000 m/s.

In this study, the seismic faults where the maximum credible earthquake (MCE) may occur are set up every 1 km in the two potential seismic zones for the consideration of the fault location uncertainty, as shown in Fig. S2. The Yaziba fault is a reverse fault, of which the strike and dip are 180° and 70°, respectively. The Leibo fault is a strike-slip fault with the strike of 225° and the dip of 60°. According to the empirical scaling law for earthquake source[2], the length and width of the seismic fault are set to 55 km and 40 km for scenarios of MCE (Mw7.5) at the Yaziba fault; the length and width of

the seismic fault are set to 45 km and 20 km for scenarios of MCE (*M*w7.0) at the Leibo fault. The multidimension source models in this study are composed of 4 layers, and parameters of them are listed in Table S1.

Table S2 presents the distribution ranges of the source process parameters for earthquake scenarios at the Yaziba fault and the Leibo fault. For the generated earthquake scenarios, the hypocenters of them can be anywhere on the fault ruptures of earthquake scenarios. Based on the constraints on the slip, rupture velocity, rise time, and rake angle ranges in the source inversion study[6] and the stochastic source process model[7], the source parameter ranges of the earthquake scenarios are defined in this study. The slip amount of each subfault is allowed to vary from 0 to 4 times the average slip value for the earthquake scenarios, while the average of them remains constant, i.e., 1.68 m for the Yaziba fault and 0.75 m for the Leibo fault. By solving the Eikonal equation, the rupture time of each subfault is derived from the distribution of rupture velocity that varies from 2.5 km/s to 3.5 km/s. The rise time of each subfault is allowed to vary from 1 s to 4 s for the Yaziba fault and 0.5 s to 2.5 s for the Leibo fault, while the averages of them are equal to 2.5 s for the Yaziba fault and 1.5 s for the Leibo fault, respectively. According to the fault exploration results, the Yaziba fault is a reverse fault while the Leibo fault is a strike-slip fault. To determine the rake, the average rake is firstly allowed to vary from 70° to 110° for the Yaziba fault and from 160° to 200° for the Leibo fault. Next the rake of each subfault is allowed to vary in the range of ±30° around the average rake.

Results

The seismic hazards for the spectral accelerations at each individual seismic fault in the potential seismic zones are presented in Fig. S3 and S4. Generally, the spectral accelerations decrease as the distance from the fault to the site increases. For the same hazard level, e.g., the exceeding probability of 50%, the PGA for the nearest seismic fault (F0) is approximately 50% higher than that for the farthest seismic fault (F10).

Therefore, the uncertainty of fault locations has non-negligible effects on the intensity of ground motions at the near-fault sites, and should be paid special attention in the seismic hazard evaluations for the target site.

Compared to traditional seismic hazard analysis methods, our method can provide hazard results for any intensity measures (IMs). Therefore, for major engineering structures such as dams, nuclear power plants, long-span bridges, and high-rise buildings, this method can give personalized seismic hazards by using optimal IMs to provide more accurate seismic hazard evaluations. Herein, four representative IMs (root-mean-square of acceleration (a_{RMS}), root-mean-square of velocity (v_{RMS}), Arias intensity (Ia), and cumulative absolute velocity (CAV)) are adopted to analyze the MCE hazards. These IMs are defined as follows,

$$\mathrm{a_{RMS}} = \left(\frac{1}{\mathrm{t_D}}\int_0^{\mathrm{t_D}} \mathrm{a(t)^2 dt}\right)^{1/2} \quad \text{(S1)}$$

where $\mathrm{t_D}$ is the total duration of ground motions, and $\mathrm{a(t)}$ is the acceleration time series.

$$v_{\mathrm{RMS}} = \left(\frac{1}{\mathrm{t_D}}\int_0^{\mathrm{t_D}} v\mathrm{(t)^2 dt}\right)^{1/2} \quad \text{(S2)}$$

where $v\mathrm{(t)}$ is the velocity time series.

$$\mathrm{CAV} = \int_0^{\mathrm{t_D}} |\mathrm{a(t)}|\, \mathrm{dt} \quad \text{(S3)}$$

$$\mathrm{I_a} = \frac{\pi}{2\mathrm{g}}\int_0^{\mathrm{t_D}} \left(\mathrm{a(t)}\right)^2 \mathrm{dt} \quad \text{(S4)}$$

Seismic hazards for the four representative IMs at the Xiluodu dam site for earthquake scenarios occurred in the Yaziba seismic zone and the Leibo seismic zone are presented in Fig. S5 and S6, respectively. In contrast to the seismic hazards represented by the spectral accelerations (Fig. 2), which are comparable for the two seismic zones, the four IMs at the river-parallel direction for the Yaziba seismic zone

are obviously higher than those for the Leibo seismic zone. It is demonstrated that the seismic hazards represented by different IMs may have significant differences for the same site. Therefore, adopting the optimal IM for different engineering structures can provide more reasonable seismic hazard evaluations.

In order to explore the spatial variations within the ground-motion field, 5 scenarios for each of 3 representative seismic hazard levels (i.e., exceedance probabilities of 10%, 50%, and 90%) are derived based on the obtained seismic hazard results. These scenarios occurring at the main rupture (F5 in Fig. S2) of both the Yaziba and Leibo faults are then simulated, yielding a total of 30 scenarios (15 scenarios for each rupture). ShakeMaps for Peak Ground Acceleration (PGA) of these scenarios are subsequently produced through forward simulations. We then compute the average (Fig. S7), standard deviation (Fig. S8), and variation coefficient (Fig. S9) for the ShakeMaps of these earthquake scenarios at both the Yaziba and Leibo faults. From Fig. S7-S9, the intensity of ground motions gradually decreases from the upper edge of the fault outward, but exhibits complex spatial distributions in the model domain. In particular, consistent with previous research [8], ground motions can be significantly amplified at the tops of hills and ridges, while it may decrease at the valleys. Additionally, the distribution of standard deviation and variation coefficient reveals much greater variability of the ground-motion intensity in the near-fault regions for different earthquake scenarios, indicating that the rupture process of earthquake scenarios has significant effects on the ground motions in regions close to the faults. Therefore, the influence of the earthquake rupture process should be paid special attention in the evaluations of seismic hazard for the near-fault regions.

Table S1 Parameters of the multidimension source models for earthquake scenarios at the Yaziba fault and the Leibo fault.

Fault name	Layer No	Subsource size (km)	Proportion coefficient for *M*	Maximum rise time (s)	Minimum rise time (s)
Yaziba	Layer 1	5	0.516	4	1
	Layer 2	2.5	0.258	2	0.5
	Layer 3	1.25	0.129	1	0.25
	Layer 4	0.625	0.065	0.5	0.125
Leibo	Layer 1	5	0.516	2.5	0.5
	Layer 2	2.5	0.258	1.25	0.25
	Layer 3	1.25	0.129	0.625	0.125
	Layer 4	0.625	0.065	0.3125	0.0625

Table S2 Distribution range of the source process parameters for earthquake scenarios at the Yaziba fault and the Leibo fault.

Fault name	Parameter	Distribution range	Constraint
Yaziba	Hypocenter	All locations on the fault	——
	Slip (m)	0-6.72	Average=1.68
	Rupture velocity (km/s)	2.5-3.5	——
	Rise time (s)	1-4	Average=2.5
	Rake (deg)	40-140	70≤Average≤110
Leibo	Hypocenter	All locations on the fault	——
	Slip (m)	0-3.0	Average=0.75
	Rupture velocity (km/s)	2.5-3.5	——
	Rise time (s)	0.5-2.5	Average=1.5
	Rake (deg)	130-230	160≤Average≤200

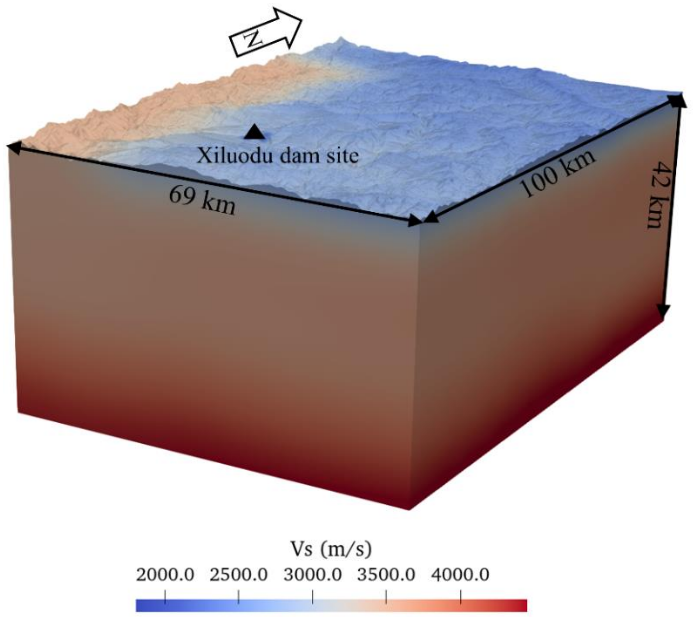

Fig. S1 Velocity structures around the Xiluodu dam site. The shear wave velocity in the model domain is presented by different colors as shown in the color bar. The black triangle indicates the Xiluodu dam site.

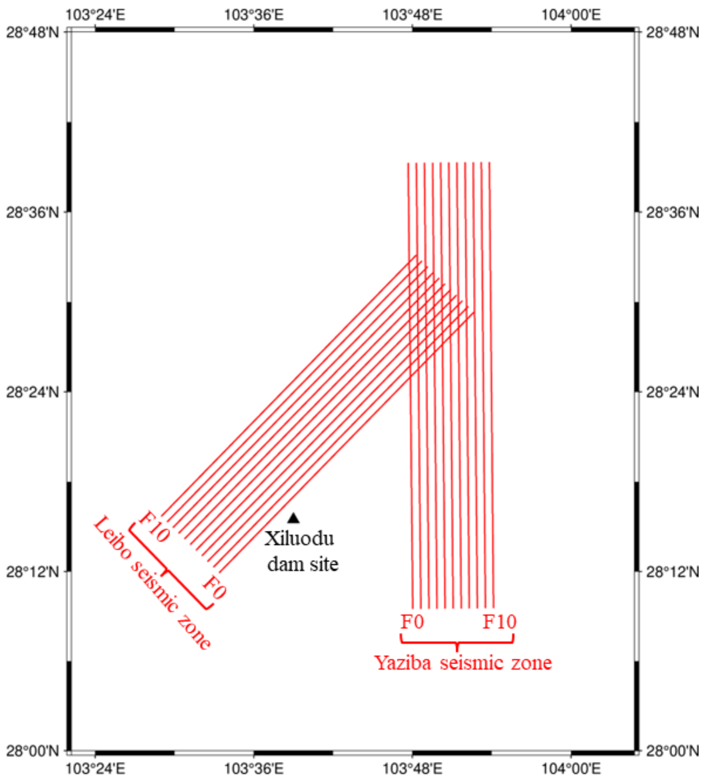

Fig. S2 Locations of the fault ruptures in the seismic zones. The fault ruptures of the maximum credible earthquakes (MCEs) are set up every 1 km in the seismic zones of Yaziba and Leibo. The red solid lines indicate the upper edges of the fault ruptures.

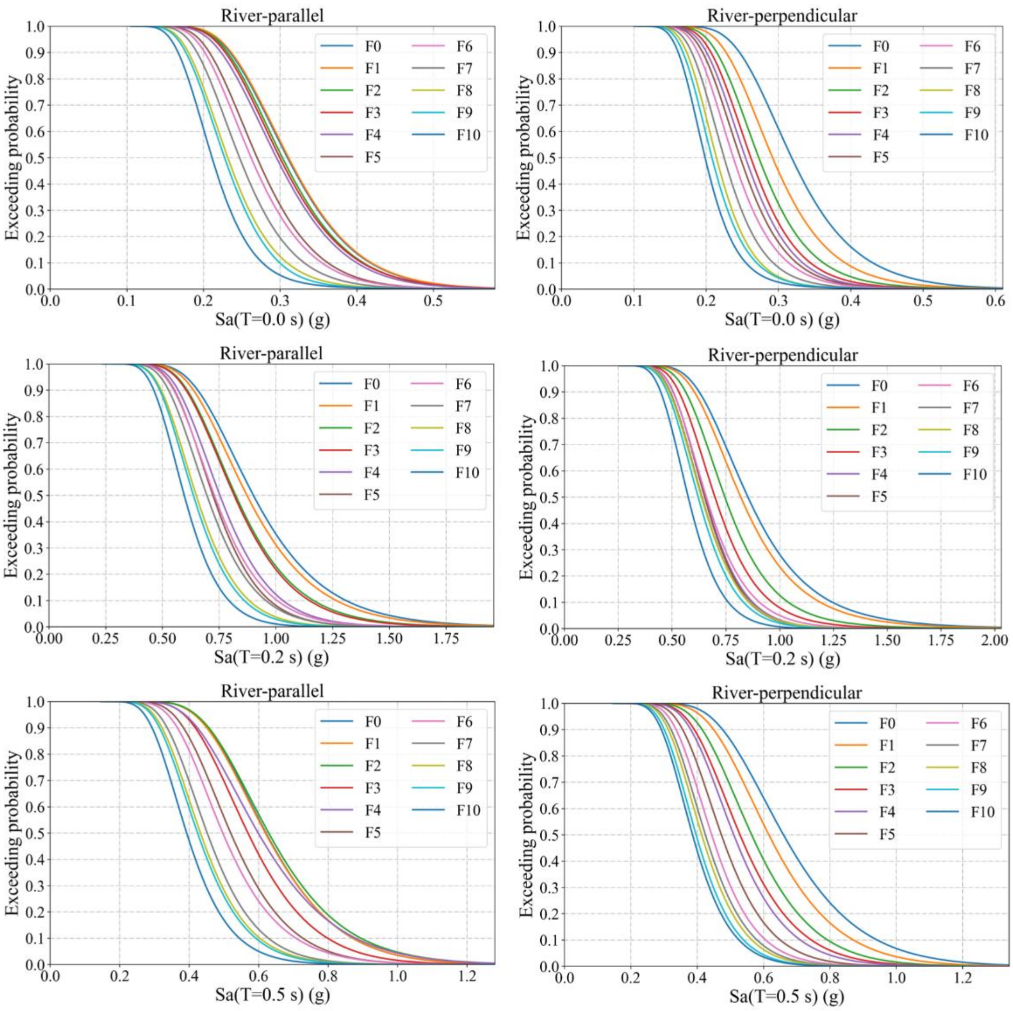

Fig. S3 Seismic hazards for spectral accelerations at the Xiluodu dam site for earthquake scenarios occurred at different fault locations in the Yaziba seismic zone. Spectral accelerations of all the maximum credible earthquake (MCE) scenarios at each individual fault are summarized to obtain the seismic hazard curves at the specified periods. The seismic hazard curves in the river-parallel direction and the river-perpendicular direction are presented for the Yaziba seismic zone.

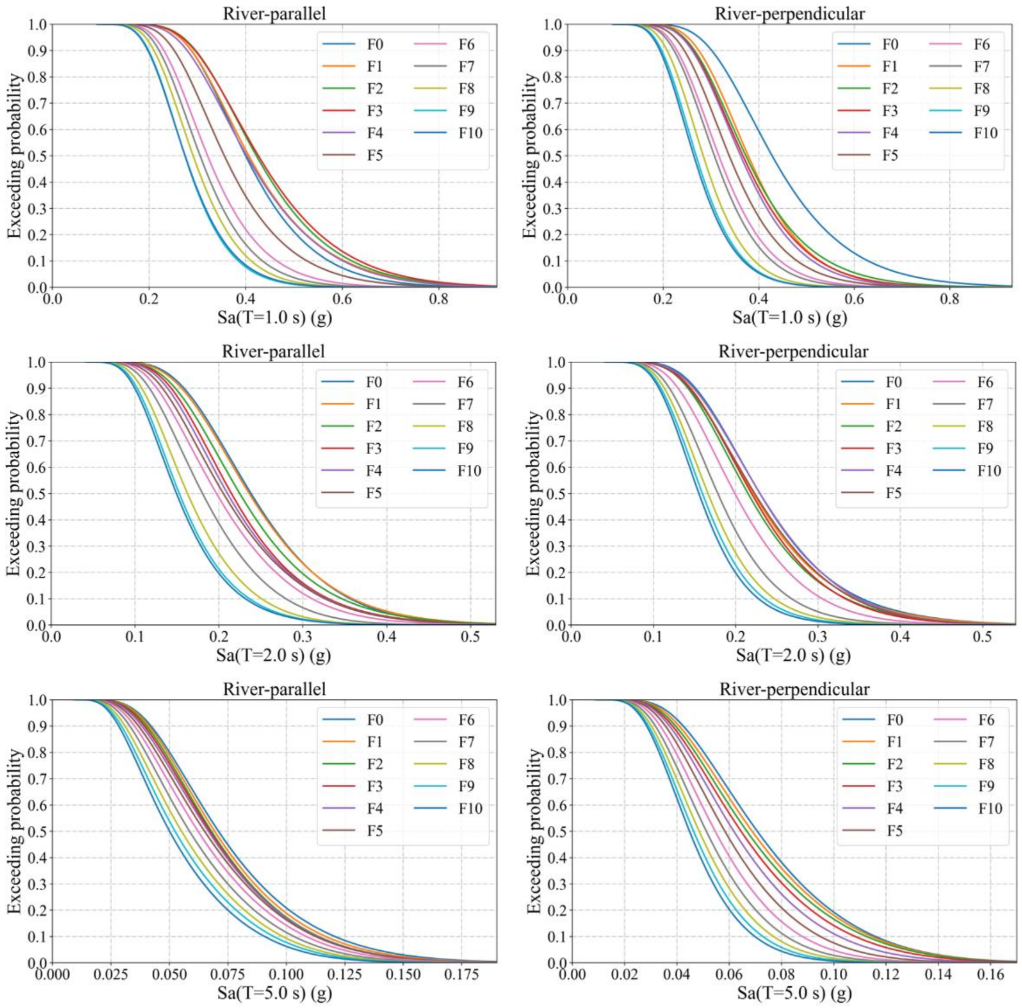

Fig. S3 Continued

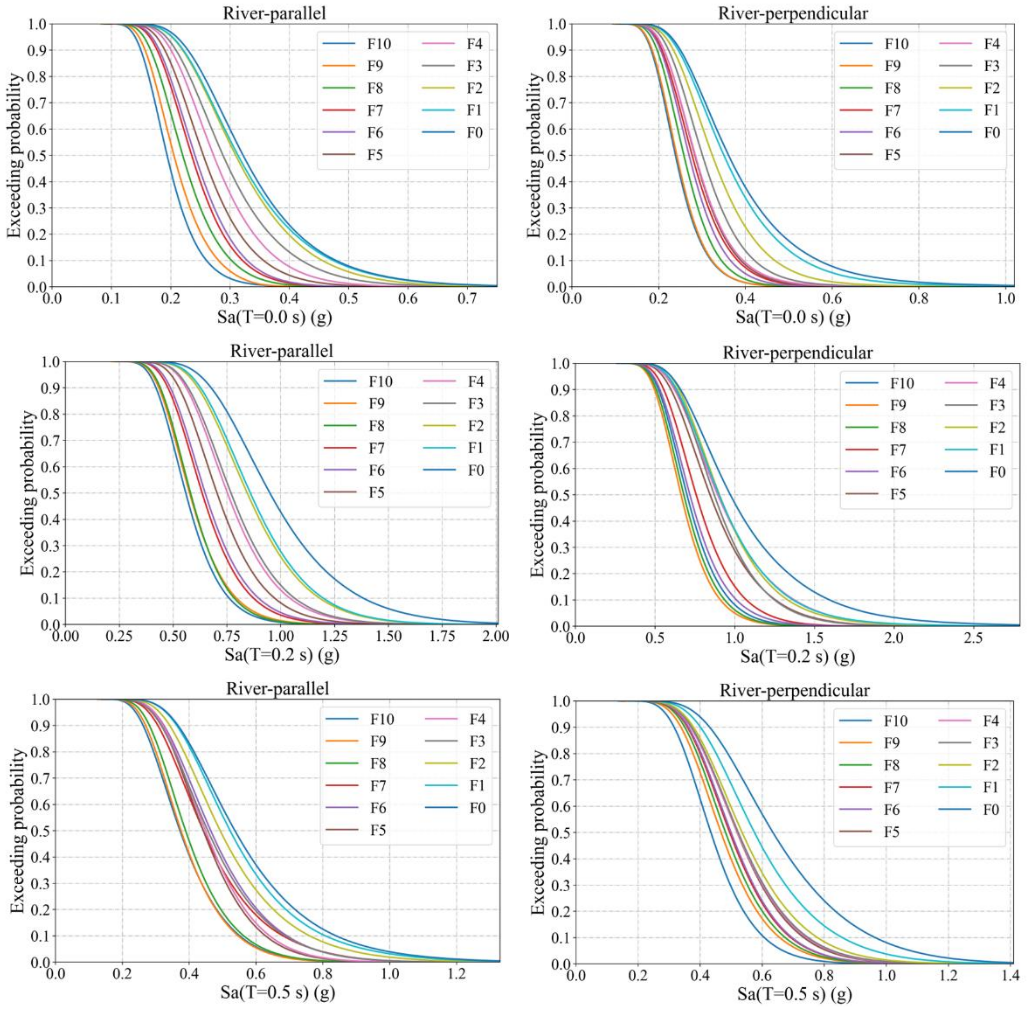

Fig. S4 Seismic hazards for spectral accelerations at the Xiluodu dam site for earthquake scenarios occurred at different fault locations in the Leibo seismic zone. Spectral accelerations of all the maximum credible earthquake (MCE) scenarios at each individual fault are summarized to obtain the seismic hazard curves at the specified periods. The seismic hazard curves in the river-parallel direction and the river-perpendicular direction are presented for the Leibo seismic zone.

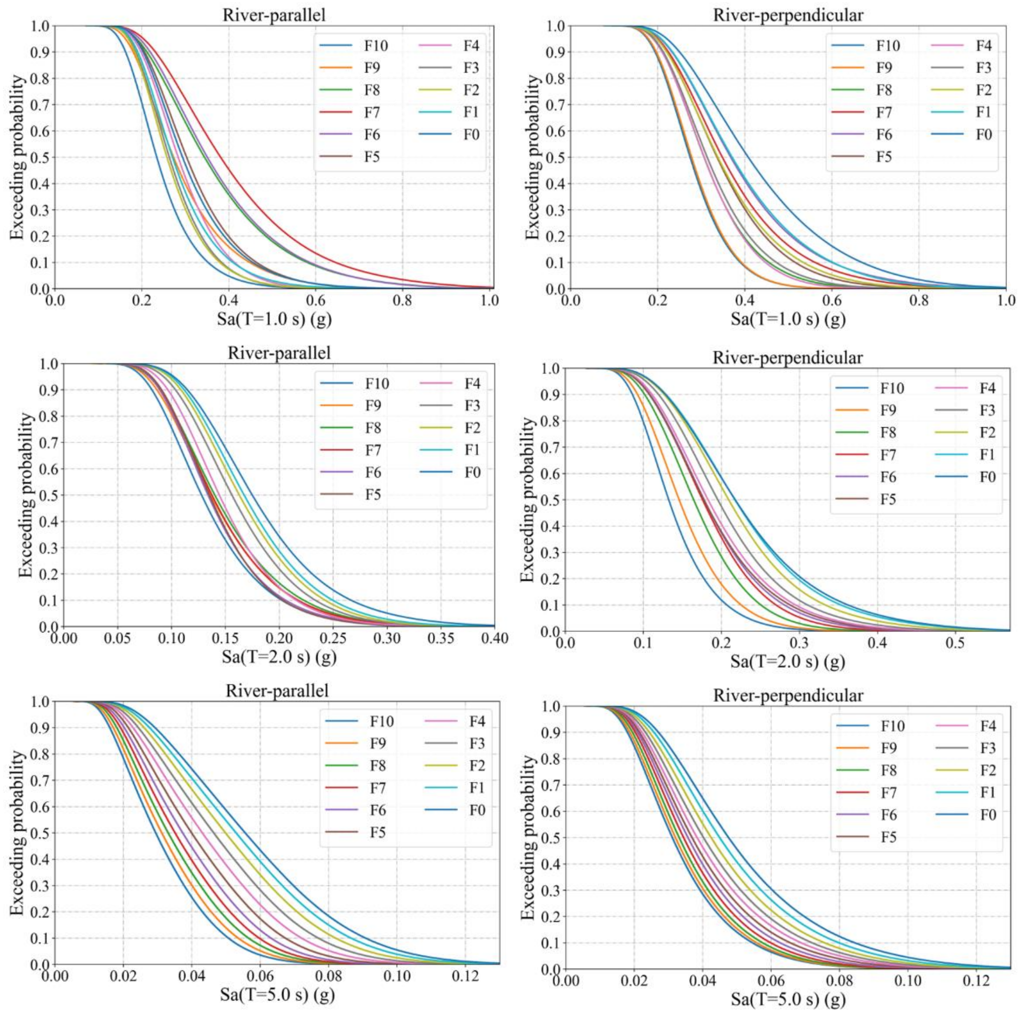

Fig. S4 Continued

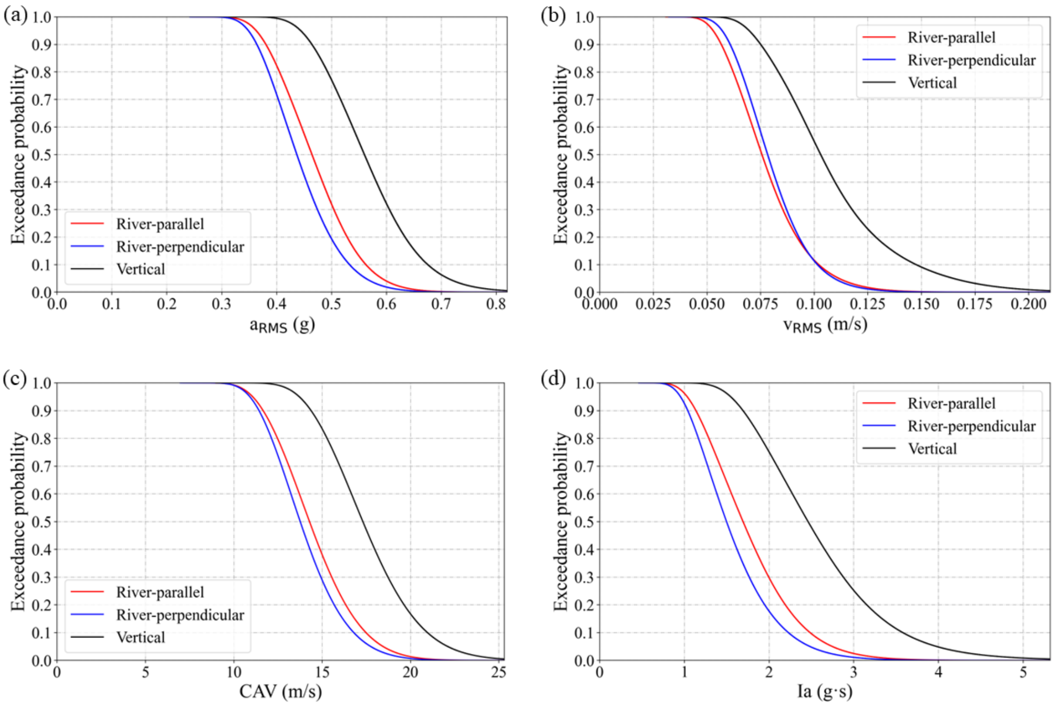

Fig. S5 Seismic hazards represented by four different intensity measures (IMs) for earthquake scenarios occurred in the Yaziba seismic zone. a Seismic hazard for the root-mean-square of acceleration (a_{RMS}) at the Xiluodu dam site for earthquake scenarios occurred in the Yaziba seismic zone. **b** Seismic hazard for the root-mean-square of velocity (v_{RMS}) at the Xiluodu dam site for earthquake scenarios occurred in the Yaziba seismic zone. **c** Seismic hazard for the cumulative absolute velocity (CAV) at the Xiluodu dam site for earthquake scenarios occurred in the Yaziba seismic zone. **d** Seismic hazard for the Arias intensity (Ia)at the Xiluodu dam site for earthquake scenarios occurred in the Yaziba seismic zone. The four IMs corresponding to all MCE scenarios are summarized to obtain the seismic hazards at the Xiluodu dam site. The seismic hazards in the river-parallel direction, river-perpendicular direction, and vertical direction are presented for the Yaziba seismic zone.

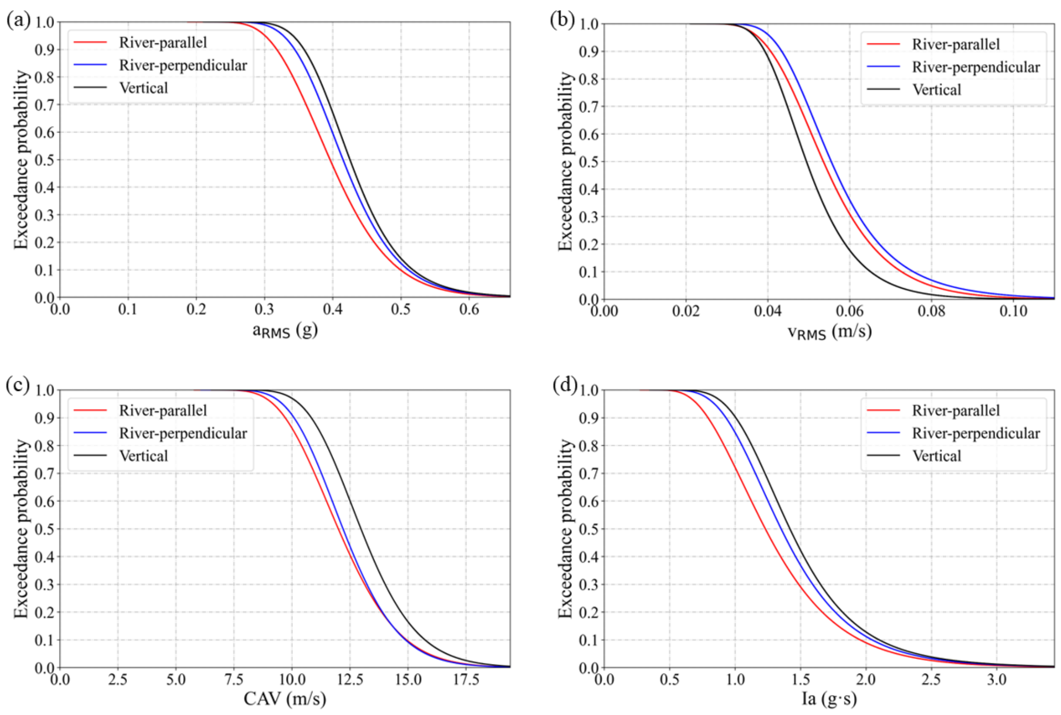

Fig. S6 Seismic hazards represented by four different intensity measures (IMs) for earthquake scenarios occurred in the Leibo seismic zone. a Seismic hazard for the root-mean-square of acceleration (a_{RMS}) at the Xiluodu dam site for earthquake scenarios occurred in the Leibo seismic zone. **b** Seismic hazard for the root-mean-square of velocity (v_{RMS}) at the Xiluodu dam site for earthquake scenarios occurred in the Leibo seismic zone. **c** Seismic hazard for the cumulative absolute velocity (CAV) at the Xiluodu dam site for earthquake scenarios occurred in the Leibo seismic zone. **d** Seismic hazard for the Arias intensity (Ia)at the Xiluodu dam site for earthquake scenarios occurred in the Leibo seismic zone. The four IMs corresponding to all MCE scenarios are summarized to obtain the seismic hazards at the Xiluodu dam site. The seismic hazards in the river-parallel direction, river-perpendicular direction, and vertical direction are presented for the Leibo seismic zone.

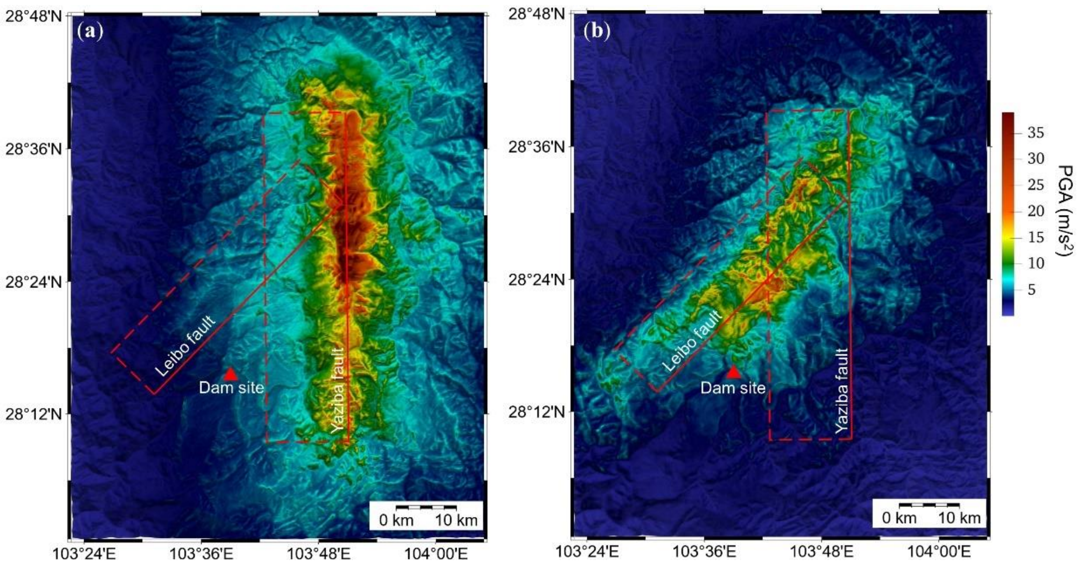

Fig. S7 Average of the ShakeMaps for Peak Ground Acceleration (PGA). a Average of the ShakeMaps for PGA of the spatial ground-motion fields generated by 15 earthquake scenarios at the Yaziba fault (F5 of the Yaziba seismic zone in Figure S2). **b** Average of the ShakeMaps for PGA of the spatial ground-motion fields generated by 15 earthquake scenarios at the Leibo fault (F5 of the Leibo seismic zone in Figure S2). The average of PGA in the model domain are presented by different colors. The red triangle represents the dam site. The top edges of the fault planes are indicated by the red solid lines, while the bottom edges of them are represented by the red dotted lines.

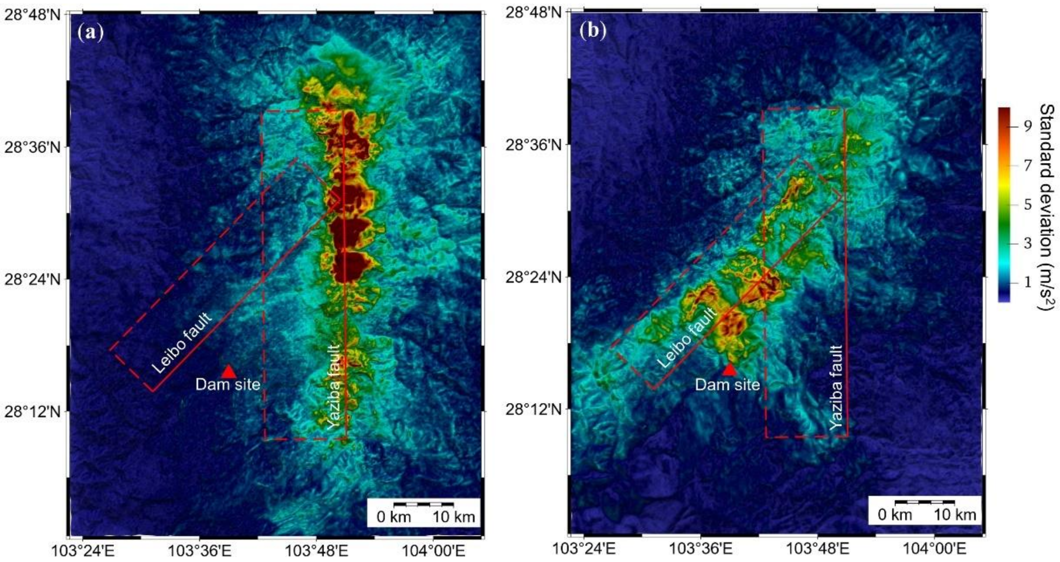

Fig. S8 Standard deviation of the ShakeMaps for Peak Ground Acceleration (PGA). a Standard deviation of the ShakeMaps for PGA of the spatial ground-motion fields generated by 15 earthquake scenarios at the Yaziba fault (F5 of the Yaziba seismic zone in Figure S2). **b** Standard deviation of the ShakeMaps for PGA of the spatial ground-motion fields generated by 15 earthquake scenarios at the Leibo fault (F5 of the Leibo seismic zone in Figure S2). The standard deviation of PGA in the model domain are presented by different colors. The red triangle represents the dam site. The top edges of the fault planes are indicated by the red solid lines, while the bottom edges of them are represented by the red dotted lines.

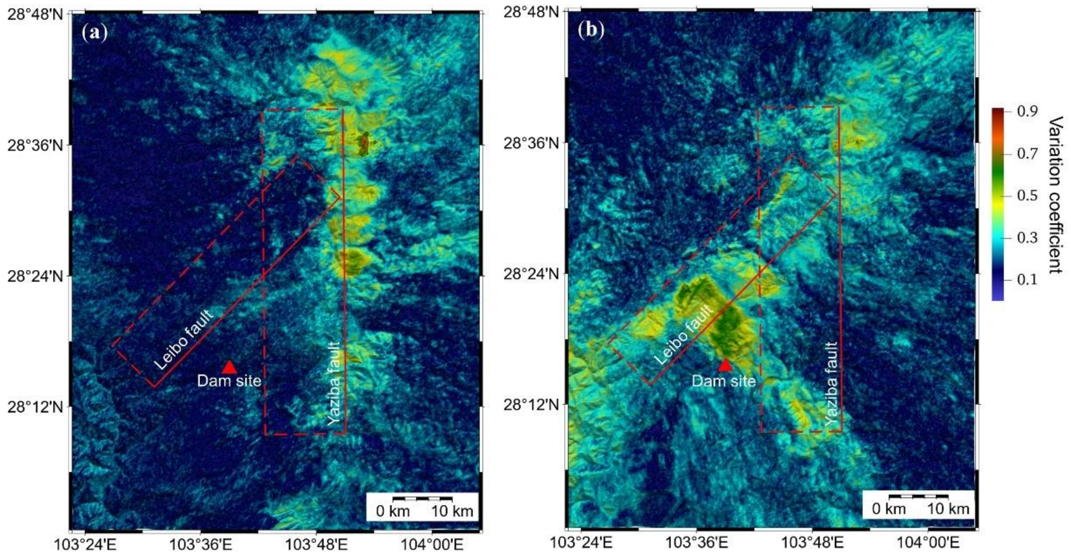

Fig. S9 Variation coefficient of the ShakeMaps for Peak Ground Acceleration (PGA). a Variation coefficient of the ShakeMaps for PGA of the spatial ground-motion fields generated by 15 earthquake scenarios at the Yaziba fault (F5 of the Yaziba seismic zone in Figure S2). **b** Variation coefficient of the ShakeMaps for PGA of the spatial ground-motion fields generated by 15 earthquake scenarios at the Leibo fault (F5 of the Leibo seismic zone in Figure S2). The Variation coefficient of PGA in the model domain are presented by different colors. The red triangle represents the dam site. The top edges of the fault planes are indicated by the red solid lines, while the bottom edges of them are represented by the red dotted lines.

Reference:

1. Wang, X.-C., Wang, J.-T., Zhang, L. & He, C.-H. Broadband ground-motion simulations by coupling regional velocity structures with the geophysical information of specific sites. *Soil Dynamics and Earthquake Engineering* **145**, 106695 (2021).
2. Thingbaijam, K. K. S., Martin Mai, P. & Goda, K. New Empirical Earthquake Source-Scaling Laws. *Bulletin of the Seismological Society of America* **107**, 2225–2246 (2017).
3. Somerville, P. *et al.* Characterizing Crustal Earthquake Slip Models for the Prediction of Strong Ground Motion. *Seismological Research Letters* **70**, 59–80 (1999).
4. Mai, P. M. Hypocenter Locations in Finite-Source Rupture Models. *Bulletin of the Seismological Society of America* **95**, 965–980 (2005).
5. Melgar, D. & Hayes, G. P. The Correlation Lengths and Hypocentral Positions of Great Earthquakes. *Bulletin of the Seismological Society of America* **109**, 2582–2593 (2019).
6. Ji, C. Source Description of the 1999 Hector Mine, California, Earthquake, Part I: Wavelet Domain Inversion Theory and Resolution Analysis. *Bulletin of the Seismological Society of America* **92**, 1192–1207 (2002).
7. Graves, R. W. & Pitarka, A. Broadband Ground-Motion Simulation Using a Hybrid Approach. *Bulletin of the Seismological Society of America* **100**, 2095–2123 (2010).

8. Lee, S.-J., Chan, Y.-C., Komatitsch, D., Huang, B.-S. & Tromp, J. Effects of Realistic Surface Topography on Seismic Ground Motion in the Yangminshan Region of Taiwan Based Upon the Spectral-Element Method and LiDAR DTM. *Bulletin of the Seismological Society of America* **99**, 681–693 (2009).
